# Heat Shock Proteins 90 kDa: Immunomodulators and Adjuvants in Vaccine Design Against Infectious Diseases

**DOI:** 10.3389/fbioe.2020.622186

**Published:** 2021-01-20

**Authors:** Mariana G. Corigliano, Valeria A. Sander, Edwin F. Sánchez López, Víctor A. Ramos Duarte, Luisa F. Mendoza Morales, Sergio O. Angel, Marina Clemente

**Affiliations:** ^1^Unidad Biotecnológica 6-UB6, Laboratorio de Molecular Farming y Vacunas, INTECH, UNSAM-CONICET, Chascomús, Argentina; ^2^Unidad Biotecnológica 2-UB2, Laboratorio de Parasitología Molecular, INTECH, UNSAM-CONICET, Chascomús, Argentina

**Keywords:** Hsp90, gp96, GRP94, chaperones, cross-presentation, re-presentation, carrier, MHC class I

## Abstract

Heat shock proteins 90 kDa (Hsp90s) were originally identified as stress-responsive proteins and described to participate in several homeostatic processes. Additionally, extracellular Hsp90s have the ability to bind to surface receptors and activate cellular functions related to immune response (cytokine secretion, cell maturation, and antigen presentation), making them very attractive to be studied as immunomodulators. In this context, Hsp90s are proposed as new adjuvants in the design of novel vaccine formulations that require the induction of a cell-mediated immune response to prevent infectious diseases. In this review, we summarized the adjuvant properties of Hsp90s when they are either alone, complexed, or fused to a peptide to add light to the knowledge of Hsp90s as carriers and adjuvants in the design of vaccines against infectious diseases. Besides, we also discuss the mechanisms by which Hsp90s activate and modulate professional antigen-presenting cells.

## Introduction

The heat shock proteins of 90 kDa (Hsp90) constitute a highly conserved protein family that is present in all living organisms (Picard, 2002; Srivastava, 2002). They are one of the most abundant heat shock proteins and represent 1–2% of the total cellular protein under healthy conditions, and their intracellular concentration increases two to three times under external stresses (Csermely et al., 1998; Srivastava, 2002). In eukaryotic cells, several Hsp90 isoforms were identified in different intracellular compartments cytosol/nucleus, endoplasmic reticulum (ER), and organelles (mitochondria and chloroplast) (Johnson, 2012). They are induced by heat shock or compartment-specific stress response signaling circuits (Heike et al., 1996; Nollen and Morimoto, 2002; Bickel and Gohlke, 2019).

In general, the main role of constitutively expressed Hsp90 is associated with their function as molecular chaperones (Prohászka and Füst, 2004; Schopf et al., 2017). Hsp90 can recognize and bind to newly synthesized and partially folded polypeptides to avoid their incorrect folding and aggregation (Pearl and Prodromou, 2006; Colaco et al., 2013). Likewise, Hsp90s are associated with the trafficking through the plasma membrane, DNA replication, signal transduction, stabilization, and activation of a great number of client proteins, which play essential roles in constitutive cell signaling and also in adaptive responses to stress (Hartl, 1996; Sangster et al., 2007; Prassinos et al., 2008; Wandinger et al., 2008; Schopf et al., 2017). Protein homeostasis (proteostasis) stress is responded by the transcription factor heat shock factor 1 (Hsf1), which is conserved in organisms ranging from yeast to human. Released and activated Hsf1 triggers a negative feedback loop by inducing the expression of chaperones Hsp70 and Hsp90 (Masser et al., 2020). Besides, under environmental stresses (heat shock, exposure to metals, or UV radiation), infectious diseases, or bacterial toxins or physiological stresses (growth factor deprivation, cell differentiation, hormonal stimulation, tissue development, fever, malignant tumors, inflammation, or autoimmunity), the Hsp90 synthesis is markedly increased to control cell homeostasis, growth, proliferation, differentiation, and cell death (Moseley, 1998; Mayer and Le Breton, 2015, Prodromou, 2016).

More than 200 protein interactors of Hsp90 have already been identified in mammals. The current list of client proteins includes cytoplasmic and nuclear receptors, transcription factors, kinases, and other unrelated proteins, sharing not common features in terms of sequence or structure [see http://www.picard.ch/Hsp90Int/index.php; (Echeverría et al., 2011)]. Most of the Hsp90 clients are essential for the biogenesis and the maintenance of numerous cellular proteins that control cell physiology, whereas others were identified as oncoproteins, tumor suppressors, proteins linked to tumor progression, invasiveness, and metastatic potential (Schopf et al., 2017; Radli and Rüdiger, 2018). Therefore, Hsp90 initially appeared as a promising target in the development of new anticancer therapies (Rivoltini et al., 2003; Nicchitta et al., 2004; Rosser et al., 2004; Joly et al., 2010). Also, Hsp90/peptide complexes purified from tumors (particularly ER Hsp90 resident: the glycoprotein 96 [gp96]) showed to be effective when employed as therapeutic vaccines in several animal models, producing an antitumoral immune response, including tumor rejection and inhibition of metastatic tumor progression (Srivastava et al., 1998). The efficient immune response against cancer cells was attributed to gp96, and it was directly associated with the antigen presentation pathway, especially that involving major histocompatibility complex class I (MHC I) molecules (Murshid et al., 2010; Ciocca et al., 2013; Weng et al., 2013). In addition, the antigen presentation capacity of Hsp90 made these chaperones promising molecules as vaccine adjuvants for a broad spectrum of pathogens (Segal et al., 2006; Udono et al., 2009; Colaco et al., 2013; Zachova et al., 2016; Sander et al., 2020).

Up to now, several efforts were made to enhance vaccine immunogenicity. Suitable adjuvants, target antigens, and carriers for delivery are needed, especially in veterinary health, where animal vaccine adjuvants and carriers are limited. It is necessary to formulate novel strategies to develop non-toxic adjuvants and less immunogenic vaccine delivery carriers to prevent various animal diseases. Several host-, pathogen-, and non–pathogen-derived Hsp90 were exploited as adjuvants with promising results (Echeverría et al., 2006; Mohit et al., 2011; Wang S. et al., 2011; Ju et al., 2014; Albarracín et al., 2015; Niu et al., 2016; Zhu et al., 2016; Bengoa Luoni et al., 2019; Sánchez López et al., 2019). This review presents a comprehensive revision of the adjuvant properties of Hsp90s and their potential application in the adjuvant design of vaccines to prevent infectious diseases. Besides, we propose mechanisms by which Hsp90s activate and modulate professional antigen-presenting cells (pAPCs) and provide their adjuvant effects based on the data reviewed.

## Hsp90 as Adjuvants in the Design of Vaccines Against Infectious Diseases

One of the most important challenges in the design of vaccines is to avoid infections caused by pathogens that have a life cycle involving an intracellular stage as many of them, including viruses and intracellular parasites, have evolved to evade the host immune system. As a consequence, hosts generated strategies that require interferon γ (IFN-γ) secretion and T-cell activation and differentiation for pathogen eradication (Leroux-Roels, 2010; Garlapati, 2012; Di Pasquale et al., 2015). Currently, there is no adjuvant strong enough to promote a T-helper 1 cell (Th1) response and to fully eliminate pathogen infections (Reed et al., 2013; Moyle, 2017; Sander et al., 2020). According to the extensively studied immunomodulatory properties of Hsp90s, it is expected that these chaperones can contribute importantly to improve the vaccine development against infectious diseases, especially against intracellular pathogens. In fact, Hsp90s from different sources have demonstrated to be potent adjuvants, generating an appropriate immune response against infectious diseases (Rapp and Kaufmann, 2004; Echeverría et al., 2006; Lee et al., 2011; Mohit et al., 2011; Wang Y. et al., 2011; Ju et al., 2014; Albarracín et al., 2015; Tan et al., 2015; Niu et al., 2016; Zhu et al., 2016; Bengoa Luoni et al., 2019; Sánchez López et al., 2019). Different strategies, including fusion protein, peptide/Hsp90 complex, and a mixture of peptides + Hsp90, were employed to evaluate the adjuvant properties of Hsp90s, and they were assayed as both DNA vaccines and recombinant protein vaccines in different animal models. These approaches take advantage of the combined adjuvant and antigen delivery capacity of Hsp90 against a wide range of antigens from a broad spectrum of intracellular pathogens (summarized in Table 1).

**Table 1:** Hsp90 from different sources used as adjuvants in vaccine formulations against intracellular pathogens.

**Adjuvant strategy**	**Type of vaccine**	**Hsp90 adjuvant**	**Antigen**	**Pathogen**	**Model**	**Immunization schedule**	**Dose/Route**	**Immune Responses**	**Protection**	**References**
N-terminal fusion protein	Recombinant protein vaccine	*E. coli-*expressed *Leishmania infantum* Hsp83	MBP	Reporter antigen	BALB/c and CF1 mice	2 doses (0, 21 d.p.i.)	50 pmol/i.p. 3 μg/i.p.	Induction of IgG1 and IgG2a; Lymphocyte proliferation; IFN-γ production	n.c.	Rico et al., 1999; Echeverría et al., 2001
N-terminal fusion protein	Recombinant protein vaccine	*E. coli-*expressed *Leishmania infantum* Hsp83	Rop2	*Toxoplasma gondii*	C57BL/6 and C3H mice	4 doses (0, 21, 34, 45 d.p.i.)	3 μg/s.c.	Induction of IgG2a and IgG2c; IFN-γ production	Higher survival rate and lower cyst load after challenge	Echeverría et al., 2006
N-terminal fusion protein	Recombinant protein vaccine	*E. coli*-expressed *Nicotiana benthamiana* Hsp90.3	MBP	Reporter antigen	BALB/c mice	2 doses (0, 21 d.p.i.)	3 μg/i.p.	Induction of IgG2a; l Lymphocyte proliferation;IFN-γ production; CD8^+^ T cells enhancement	n.c.	Corigliano et al., 2013
N-terminal fusion protein	Recombinant protein vaccine	*E. coli*-expressed N-terminal domain of *Xenopus laevis* gp96N_1−355_	E7 antigen	Human Papillomavirus type 16	C57BL/6 mice	2 doses (0, 21 d.p.i.)	200 pmol/s.c.	Induction of IgG1 and IgG2a; IFN-γ production	Delay of tumor growth	Mohit et al., 2011
C-terminal fusion protein	Recombinant protein vaccine	*E. coli*-expressed *Mus musculus* gp96	OVA and β-GAL peptides	Reporter antigens	BALB/c and C57BL6 mice	n.i.	30 μg/i.p.	Induction of TNF-α and IFN-γ	n.c.	Moré et al., 1999
C-terminal fusion protein	Recombinant protein vaccine	Plant-expressed *Leishmania infantum* Hsp83	SAG1	*Toxoplasma gondii*	C57BL/6 mice	5 doses (0, 7, 14, 21, 28 d.p.i)	4–6 μg/oral	Induction of total IgG	Lower cyst load after challenge	Albarracín et al., 2015
C-terminal fusion protein	Recombinant protein vaccine	*E. coli*-expressed *Nicotiana benthamiana* Hsp90.3	B- and T-cell SAG1 peptide	*Toxoplasma gondii*	C57BL/6 mice	4 doses (0, 14, 28, 42 d.p.i)	9 μg/i.p.	Induction of IgG2a; IFN-γ production	Lower cyst load after challenge	Sánchez López et al., 2019
C-terminal fusion protein	DNA vaccine	*Mus musculus* gp96	MHC epitopes of p60_217−225_ and LLO_91−99_	*Listeria monocytogenes*	BALB/c mice	3 doses (0, 7, 28 d.p.i)	50 μg/i.m.	IFN-γ production; CD8^+^ T cells enhancement	Higher survival rate	Rapp and Kaufmann, 2004
C-terminal fusion protein	DNA vaccine	N-terminal domain of *Mus musculus* gp96N_1−355_	Synthetic HBcAg_1−149_ peptide	Hepatitis B virus	BALB/c mice	3 doses (0, 21, 42 d.p.i)	100 μg/i.m.	Induction of IgG2; CD8^+^ T cells enhancement	n.d.	Yan et al., 2007
C-terminal fusion protein	DNA vaccine	NH_2_-terminal geldanamycin-binding domain of *Mus musculus* gp96	Circumsporozoite protein	*Plasmodium yoelii*	BALB/c mice	3 doses (0, 14, 28 d.p.i.)	100 μg/i.m.	Induction of total IgG; IFN-γ production; CD8^+^ T cell enhancement	Lower liver parasite burden and parasitemia	Tan et al., 2015
C- and N-terminal fusion protein	DNA vaccine	N-terminal domain of *Mus musculus* gp96N_1−338_	B- and T- cell epitopes of Core_132−142_, E2_405−414_, NS3_1073−1081_, NS5B_2727−2735_, NS3_1248−1262_ and E2_412−426_	Hepatitis C Virus	CB6F1 mice	3 doses (0, 21, 42 d.p.i)	100 μg/i.m.	Induction of IgG2a; IFN-γ and TNFα production; CD8^+^ T-cells enhancement	n.d.	Pishraft-Sabet et al., 2015
Binding complex	Recombinant protein vaccine	*E. coli*-expressed *Mus musculus* gp96N_22−355_ and *Mus musculus* gp96C_561−751_	7-mer and 9-mer peptides, HBcAg_88−94_ and HBcAg_87−95_	Hepatitis B Virus	BALB/c mice	3 doses (0, 21, 42 d.p.i.)	10 μg/s.c.	CD8^+^ T cells and CTL enhancement	n.d.	Li et al., 2005
Binding complex	Recombinant protein vaccine	Yeast*-*expressed *Mus musculus* gp96	Synthetic HBcAg_87−95_ peptide	Hepatitis B Virus	BALB/c mice	3 doses (0, 21, 42 d.p.i.)	50 μg of HBcAg_87−95_ bound to 20 μg of y-Mmgp96/s.c.	CD8^+^ T cells and CTL enhancement	n.d.	Li et al., 2011
Binding complex	Recombinant protein vaccine	Yeast*-*expressed *Mus musculus* gp96	H1N1 split-virus vaccine	Influenza A (H1N1) virus	BALB/c mice	2 doses (0, 14 d.p.i.)	H1N1 split-virus vaccine bound to 20 μg y-Mmgp96/i.p.	Induction of IgG2a; IFN-γ and TNFα production; CD8^+^ T-cells enhancement	Lower mortality rate and less viral titer after the challenge	Ju et al., 2014
Mixture	Recombinant protein vaccine	Mammal cell-expressed *Mus musculus* gp96	β-GAL	Reporter antigen	C57BL/6 mice	2 doses (0, 7 d.p.i)	1 or 10 μg of gp96 mixed with 1 or 10 μg of β-GAL/s.c.	n.d.	n.c.	Binder et al., 2007
Mixture	Recombinant protein vaccine	*E. coli*-expressed N-terminal domain of porcine gp96N_22−370_	Cp1 and Cp2 peptides	Porcine reproductive and respiratory syndrome virus	BALB/c mice and piglet	4 doses (0, 10, 20, 30 d.p.i.)	50 μg of porcine gp96N_22−370_ mixed with 50 μg of Cp1 + Cp2/i.m.	Lymphocyte proliferation and IFN-γ production; Induction of IFN-γ and IL-12 in sera	n.d.	Chen et al., 2012
Mixture	Recombinant protein vaccine	*E. coli*-expressed N-terminal domain of porcine gp96N_22−355_	Synthetic Gp4-59B, Gp5-37B, Gp4-7T, Gp4-170T, Gp5-117T, Gp5-149T, N-49T, N-63T, N-104T and Pan DR T-helper cell epitope peptides	Porcine reproductive and respiratory syndrome virus	Piglets	4 doses (0, 14, 28, 42 d.p.i)	0.5 mg of porcine gp96N_22−355_ mixed with 0.2 mg of each peptide/i.m.	Induction of total IgG; IL-12 and TNFα production	Higher protection during the first 5 days after the infection	Chen et al., 2013
Mixture	Recombinant protein vaccine	Baculovirus-expressed N-terminal domain of porcine gp96N_22−370_	PCV2Cap protein	Porcine circovirus type 2	Mice and pigs	2 doses (0, 21 d.p.i.)	750 μg of gp96N_22−370_ mixed with 750 μg of PCV2Cap protein	Induction of total IgG; Lymphocyte proliferation; IFN-γ production	Lower viral loads in blood and lymph nodes after the infection	Zhu et al., 2016
Mixture	Recombinant protein vaccine	*Pichia pastoris*-expressed N-terminal domain canine gp96N_22−355_	AR16 and hPAB synthetic peptides from N protein	Rabies virus	BALB/c mice and beagles	3 doses (0, 14, 28 d.p.i.)	500 μg of gp96N_22−355_ mixed with 250 μg of each peptide	Induction of total IgG	Higher survival rate after the infection	Niu et al., 2016
Mixture	Recombinant protein vaccine	*E. coli-*expressed *Clonorchis sinensis* Hsp90	Proline-rich (ProR) antigen	*Clonorchis sinensis*	C57BL/6 mice	3 doses (0, 14, 28 d.p.i.)	n.i.	Induction of IgG1 and IgG2a, toward IgG1	n.d.	Chung et al., 2017
Mixture	Recombinant protein vaccine	*E. coli*-expressed *Arabidospis thaliana* Hsp81.2	NcSAG1	*Neospora caninum*	BALB/c mice	2 doses (0, 14 d.p.i)	30 μg of AtHsp81 mixed with 10 μg of NcSAG1	Induction of IgG1 and IgG2a	Higher offspring survival rate and lower parasitic load after the challenge	Bengoa Luoni et al., 2019
Mixture	DNA vaccine + prime boost of recombinant proteins	*E. coli*-expressed N-and C-terminal domain of *Xenopus laevis* gp96N_22−360_ and gp96C_549−851_	E7 antigen	Human papillomavirus type16	C57BL/6 mice	2 doses (0; 21 d.p.i.)	100 μg/i.m. and s.c.	Induction of IgG1 and IgG2a, toward IgG1; Lymphocyte proliferation and IFN-γ production	n.d.	Bolhassani et al., 2008
Mixture	DNA vaccine + prime boost of recombinant proteins	*E. coli*-expressed *Mus musculus* gp96	HBcAg and HBsAg	Hepatitis B Virus	BALB/c mice	4 doses (0, 7, 14, 28 d.p.i.)	10 μg and 50 μg/i.m. and s.c.	Induction of IgG1 and IgG2a; Lymphocyte proliferation; CD8^+^ T cells enhancement	Reduction of HB levels in serum, HBc expression in liver, and viral load after infection	Wang S. et al., 2011

*d.p.i., days post-immunization; s.c., sub-cutaneous; i.m., intra-muscular; i.p., intra-peritoneal; n.c., not correspond; n.d., not determined; n.i., not indicated*.

In the following section, we will summarize and analyze the different strategies that use the ability of Hsp90/gp96 as an adjuvant to generate specific immune responses against different viruses, bacteria, and parasite antigens.

### Administration of Antigenic Peptides as a Fusion Protein or Peptide/Hsp90 Complex

The most commonly used strategy in the vaccine design based on Hsp90 or gp96 as adjuvants consist of covalent linkage or binding complexes between the antigenic peptides and these chaperones. These strategies are useful, simple, versatile, and feasible of implementation, and it has been demonstrated that they elicit an appropriate immune response against intracellular pathogens, which is more efficient than the administration of the mixture of antigen + adjuvant (Table 1). The adjuvant capacity of Hsp90s was tested with reporter antigens and also with pathogens.

#### Reporter Antigens to Study Hsp90 Adjuvanticity

An interesting first approach to analyze whether a given Hsp90 facilitates the immunomodulation or the antigen presentation is to assay it either covalently linked and/or bound to a reporter antigen or a protein with non-immunogenic characteristics, like ovalbumin (OVA), β-galactosidase (β-GAL), or the maltose-binding protein (MBP) (Moré et al., 1999; Rico et al., 1999; Echeverría et al., 2001; Binder et al., 2007; Corigliano et al., 2013). Initially, Moré et al. (1999) showed that *Escherichia coli*–expressed *Mus musculus* C-terminal end of gp96 (rMmgp96) fusion proteins rMmgp96–β-GAL and rMmgp96-OVA activate specific CTL against the reporter peptides *in vitro*. However, rMmgp96–β-GAL was not able to generate β-GAL-specific T cells in intraperitoneally (i.p.) immunized BALB/c and C57BL6 mice. As the dimeric gp96 form is connected by the C-terminal domain, the authors suggest that the addition of β-GAL peptide covalently linked to the C-terminal of rMmgp96 could inhibit the delivery of the peptide into the MHC I–related pathway, impeding the generation of CTL *in vivo* (Moré et al., 1999). Besides, the MBP was fused to the N-terminal of the pathogenic protozoan *Leishmania infantum* Hsp83 (LiHsp83). The *E. coli*–expressed MBP-LiHsp83 fusion protein (rMBP-LiHsp83) was assayed in BALB/c and CF1 mice by i.p. immunizations. The humoral immune response against the MBP antigen was significantly enhanced in rMBP-LiHsp83–immunized mice compared to MBP– or MBP+ rLiHsp83-immunized mice, showing a mixed IgG1:IgG2a immune response against MBP (Rico et al., 1999; Echeverría et al., 2001). In addition, the analysis of the cellular immune response showed that stimulation of splenocytes from rMBP-LiHsp83–immunized mice with MBP elicited lymphoproliferation and IFN-γ production (Echeverría et al., 2001) and both T cells and non–T cells from naive BALB/c mice were able to proliferate (Rico et al., 1999). More recently, Corigliano et al. (2013) performed a similar approach using *E. coli*–expressed *Nicotiana benthamiana* Hsp90.3 (rNbHsp90.3). The results showed that rNbHsp90.3 fused to MBP (rMBP-NbHsp90.3) generated a long-lasting humoral immune response with a predominance of IgG2a isotype in i.p. immunized BALB/c mice. Moreover, rMBP-NbHsp90.3–immunized mice produced high levels of IFN-γ with an increased number of CD8^+^ T cells and significantly enhanced MHC I expression levels (Corigliano et al., 2013).

#### Hsp90 as Adjuvant of Antigens From Pathogens

As the studies carried out with generic antigens do not necessarily predict the exact biological response against immunogens from pathogens such as viruses, microbes, or parasites, Hsp90s were also assayed in different models of infections. A well-known model of host–parasite interaction is toxoplasmosis. *Toxoplasma gondii*, the etiological agent of toxoplasmosis, is an intracellular protozoan parasite with human and veterinary importance. The host protective immune response against *T. gondii* infection is based on the secretion of the main humoral mediator IFN-γ, with a Th1 profile (Gazzinelli et al., 1991; Kang et al., 2000). In this sense, rLiHsp83 adjuvanticity was assayed fused to Rop2 antigen from *T. gondii* in an adjuvant-free vaccination system. The authors showed that *E. coli*–expressed Rop2-LiHsp83 (Rop2 fused to Hsp83 N-terminal end) gave a humoral immune response with increased levels of IgG2a/IgG2c isotypes in BALB/c, C57BL/6, and C3H mice. They also demonstrated that rRop2-LiHsp83–subcutaneously (s.c.) immunized mice secreted IFN-γ after *in vitro* stimulation with rRop2 (Echeverría et al., 2006). Interestingly, the challenge with *T. gondii* in rRop2-LiHsp83–immunized mice resulted in a higher survival rate and in a significantly lower cyst load compared to controls (Echeverría et al., 2006). Later, Albarracín et al. (2015) expressed the LiHsp83 fused to *T. gondii* SAG1 antigen in tobacco chloroplasts (chLiHsp83-SAG1, where SAG1 was fused to LiHsp83 C-terminal end). The authors first demonstrated that chLiHsp83 enhanced the solubility and expression of the SAG1 protein during antigen production, an extremely important aspect as plants frequently show low expression levels of heterologous proteins. Besides, they observed that C57BL/6 mice orally immunized with chLiHsp83-SAG1 elicited an important reduction of the cyst burden that correlated with an increased humoral immune response, suggesting that chLiHsp83 is a promising candidate as an oral adjuvant (Albarracín et al., 2015). More recently, Sánchez López et al. (2019) evaluated the adjuvant properties of rNbHsp90.3 using the fusion protein strategy with a peptide from *T. gondii* SAG1 protein, which carries B- and T-cell epitopes (SAG1_HC_). Similar to the strategy used by Albarracín et al. (2015), the peptide was fused to C-terminal end of rNbHsp90.3. The authors observed that the humoral immune response is addressed toward a Th1 profile with high levels of IgG2a/IgG2b and IFN-γ when the antigenic peptide is fused to and carried by rNbHsp90.3 in C57BL/6-immunized mice (Sánchez López et al., 2019). Remarkable, rNbHsp90.3-SAG1_HC_-immunized mice presented lower cyst load after challenge (Sánchez López et al., 2019).

DNA vaccines were also used in the study of Hsp90 as adjuvants. Intramuscular (i.m.) immunization of BALB/c mice with different plasmids encoding *Listeria monocytogenes* peptides fused to the C-terminal end of rMmgp96 induced a peptide-specific CTL response along with IFN-γ production and also induced efficient protection after challenge with a lethal dose of *L. monocytogenes* (Rapp and Kaufmann, 2004). Similarly, Pishraft-Sabet et al. (2015) observed that a DNA vaccine efficacy improved when the hepatitis C virus polytope (PT) antigen was fused to the N-terminal end of Mmgp96_1−338_, but not at the C-terminal end of this truncated gp96_1−338_ version. In fact, mice immunized with PT-Mmgp96N_1−338_ DNA produced a significantly higher amount of IgG2a antibody isotype compared to the fusion Mmgp96N_1−338_-PT DNA (Pishraft-Sabet et al., 2015). Therefore, they suggested that Mmgp96N_1−338_-PT fusion could produce conformational changes or steric hindrance, as well as change in the ubiquitination pattern, altering vaccine efficacy and antigen presentation.

It must be noted that the adjuvant properties present in rNbHsp90.3 and rLiHsp83 are present irrespective if the antigen of interest is fused to the N- or the C-terminal end of these chaperones. This argues against what is proposed by Moré et al. (1999), who suggested that the fusion of β-GAL to the C-terminal end of Mmgp96 could affect the dimerization of the chaperone and therefore its adjuvant capacity. On the other hand, rMmgp96_1−338_ demonstrated to maintain its adjuvant properties as DNA vaccine only when the antigen is fused to the N-terminal end. Probably, the antigenic properties of the antigen studied have a notable role in the activation of the immune response, and in each case, it should be evaluated.

#### Adjuvanticity of Hsp90 Domains

Hsp90 protein contains three well-defined domains: N-, middle, and C-terminal (Buchner, 1999; Krishna and Gloor, 2001; Kishimoto et al., 2005; Kumar et al., 2007; Kadota and Shirasu, 2012). The N-terminal domain of Hsp90 is the most conserved among the studied species (Johnson, 2012), and additional studies using the N-terminal domain of eukaryotic gp96 demonstrated that this domain has immunomodulatory activity and that residues 34–222 are sufficient to retain the bound peptide (Gidalevitz et al., 2004). Different vaccines were evaluated to clarify which domain (N- or C-terminal) from eukaryotic gp96 is involved in the induction of antigen-specific T-cell response. Some reports demonstrated that the N-terminal region of Hsp90 alone is sufficient to stimulate an immune response against the antigen of interest. Yan et al. (2007) showed the adjuvant effect of the N-terminal domain of Mmgp96 (Mmgp96N_1−355_) to enhance the potency of hepatitis B virus (HBV) DNA vaccines. They used the major S, middle S2S envelope proteins, and truncated HBcAg_1−149_ of HBV to construct fusion genes by linking the Mmgp96N_1−355_ to its N-terminus (HBcAg_1−149_-Mmgp96N_1−355_). BALB/c mice i.m. immunized with the fusion genes showed 2–6-fold higher HBV-specific CD8^+^ T cells and 20-fold higher IgG2a antibody level compared to the antigens alone, suggesting that Mmgp96N_1−355_ may be used as a molecular adjuvant to enhance the potency of DNA-based HBV vaccines (Yan et al., 2007). Similar results were found by Tan et al. (2015), who designed a novel malaria DNA vaccine by fusing the full length of circumsporozoite protein (CSP), an immunodominant antigen of the attenuated sporozoite vaccine (Kumar et al., 2007), with a fragment of the N-domain of Mmgp96 (Mmgp96-CSP). DNA-vaccinated BALB/c mice with this recombinant plasmid induced both specific antibody and CD8^+^ T-cell response, which is essential to design a highly effective antimalaria subunit vaccine. Regarding recombinant vaccines, early studies observed that murine GRP94 N-terminal region (GRP94_34−355_) is sufficient for peptide binding and for the binding to dendritic cell (DC) receptors (Biswas et al., 2006; Jockheck-Clark et al., 2010). Besides, Li et al. (2005) evaluated two synthetic 9-mer peptide and 7-mer peptide isolated from an HBV patients associated with *E. coli*–expressed Mmgp96N_22−355_ or Mmgp96C_561−751._ The peptide/gp96 complexes were used to immunize s.c. BALB/c mice. Interestingly, the rMmgp96N_22−355_ elicited a peptide-specific CTL immune response, whereas the rMmgp96C_561−751_ did not. However, the cellular immune response elicited by rMmgp96N_22−355_ was not as effective as those generated by rMmgp96 full length (Li et al., 2005). By contrast, studies on the effects of linkage of human papillomavirus type 16 (HPV16) E7 to N-terminal and C-terminal of human gp96 (Hsgp96) showed that linkage of C-terminal domain to HPV16 E7 drives humoral response toward IgG2a, suggesting that C-terminal fragment of Hsgp96 is also effective and potent in producing humoral and T cell–mediated immune responses (Bolhassani et al., 2008; Daemi et al., 2012; Pishraft-Sabet et al., 2015). In conclusion, while the N-terminal region of Hsp90/gp96 is strongly associated with the adjuvant capacity of the chaperone, the adjuvant potential of the C-terminal region remains controversial. It is worth to say that several regions along to Hsp90 can bind different client proteins (Xu et al., 2012), suggesting that the full-length chaperone could be a requirement of some antigens to be carried and presented by Hsp90 to the immune system. Up to now, the mechanism is not further elucidated.

### Coadministration of Antigenic Peptide and Hsp90 as a Mixture

Some studies showed that the Hsp90 coadministered with an antigenic peptide as a mixture not always acts as adjuvant (Blachere et al., 1997). Later, Binder et al. (2007) showed that s.c. immunization of C57BL/6 mice with a mixture of Mmgp96 and β-GAL proteins expressed in murine cells did not elicit a specific CTL response, suggesting that Mmgp96 did not confer detectable adjuvant capacity on the chaperoned antigen *in vivo*. However, other studies demonstrated that the administration of antigenic peptides + Hsp90 as a mixture can modulate the humoral and cellular immune response against, providing specific protection against those pathogens that carry the antigens used in the immunizations.

Eukaryotic gp96 was used as an adjuvant in mixed formulations to enhance antigen-specific immune responses against different virus diseases. To develop a more effective vaccine to prevent the porcine reproductive and respiratory syndrome virus (HP-PRRSV), porcine gp96 was mixed with the antigenic peptides, and the adjuvant properties of the N-terminal domain of porcine gp96 (p-gp96N) were evaluated (Chen et al., 2012, 2013). In one case, the *E. coli*–expressed p-gp96N (rp-gp96N_22−370_) was coadministered with two multiepitope subunit vaccines (Cp1 and Cp2), based on the conserved B-cell epitopes of viral proteins, in both mouse and piglet models (Chen et al., 2012). After i.m. vaccination with the mixture of Cp1 or Cp2 + rp-gp96N_22−370_, both the lymphocyte proliferation and IFN-γ responses were greatly enhanced in mice, whereas IFN-γ and interleukin 12 (IL-12) levels were significantly increased in sera of the piglets (Chen et al., 2012). In the other case, two synthetic T-cell epitopes and eight synthetic B-cell epitopes were i.m. coadministered in pigs with rp-gp96N_22−355_ (Chen et al., 2013). Sera from piglets immunized with all peptides and rp-gp96N_22−355_ produced highly specific anti-PRRSV and anti–B-cell epitopes antibodies. Besides, they showed that only T-cell peptides + rp-gp96N_22−355_ mixture elicited CTL response with IL-12 and tumor necrosis factor α (TNF-α) secretion (Chen et al., 2013). Furthermore, after challenging with HP-PRRSV, piglets i.m. vaccinated with the mixture of rp-gp96N_22−355_ + synthetic peptides showed lighter clinical signs, lower viremia, and fewer pathological lesions (Chen et al., 2013). Currently, a porcine circovirus type 2 (PCV2Cap) subunit-based vaccine is commercially available. However, its efficacy is constantly under revision, being important to improve the levels of protection against PCV2 challenge. In this context, Zhu et al. (2016) analyzed the adjuvant effect of baculovirus-expressed p-gp96N_22−370_ (bac-p-gp96N_22−370_) and p-Hsp90 (bac-p-Hsp90) in the porcine circovirus type 2 (PCV2) model. They observed that bac-Cap + bac-p-gp96N_22−370_ and bac-Cap + bac-p-Hsp90 formulations augmented the humoral immunity in mice and pigs as neutralizing antibodies levels against PCV2 were significantly increased. The cellular immunity against PCV2 was also increased, as it was observed by the significantly higher proliferation and the production of IFN-γ in both models. Remarkably, pigs showed reduced clinical signs of infection and lower viral loads in blood and lymph nodes after challenge (Zhu et al., 2016). Similarly, Niu et al. (2016) evaluate the efficacy of the administration of multiepitope-based vaccine candidates against the rabies virus (RABV) with yeast *Pichia pastoris*–expressed canine gp96N_22−355_ (yc-gp96N_22−355_) as an adjuvant. As stray dogs and wild animals are natural reservoirs of the RABV, two synthetic peptides from N protein (AR16 and hPAB), an important antigen of RABV, were evaluated in both dogs and mice models by intradermal immunizations (Niu et al., 2016). After immunization with the mixture of AR16/hPAB + yc-gp96N_22−355_, neutralizing antibodies were detected in both animal models, demonstrating that the vaccine candidates elicited a strong humoral response and immunity against virulent strain of RAVB. Besides, Wang S. et al. (2011) explored the mechanisms of Mmgp96 to mediate between CTL and regulatory T cells, which may facilitate the development of an effective gp96-based therapeutic vaccine against chronic HBV infection. They observed that a treatment based on HBcAg + rMmgp96 mixture led to decreased regulatory T cell populations, to reduce HB levels in serum and HBc expression in the liver, and also to diminish the viral load, suggesting that rMmgp96 is able to break the HBV immunotolerance (Wang S. et al., 2011). Also, rMmgp96 can induce a specific CTL and a balanced humoral immune response (IgG1:IgG2a mixed antibody production) against HBsAg and HBcAg in BALB/c-HBV mice (Wang S. et al., 2011).

On the other hand, Bolhassani et al. (2008) developed an interesting system to develop a therapeutic DNA vaccine against HPV16 that used *Xenopus laevis* gp96 (Xlgp96) as an adjuvant. The authors generated two plasmids, one expressing an HPV16 E7 protein and the other expressing Xlgp96. The coinjection of both plasmids as DNA/DNA vaccine elicited high IgG2a production and revealed a significant E7-specific IFN-γ response in mice. Besides, immunizations with DNA E7 + full-length rXlgp96 followed by a boost with rXlgp96N_22−360_ or rXlgp96C_549−851_ showed a mixed IgG1:IgG2a antibodies production, with a significantly high production of IgG1 isotype, suggesting that the different truncated forms of gp96 are capable of eliciting a mixed Th1/Th2 immune response with high intensity toward a Th2 profile (Bolhassani et al., 2008).

In addition to gp96, cytosolic Hsp90s also demonstrated adjuvant capacity when they are mixed with antigens of interest. Bengoa Luoni et al. (2019) studied the adjuvant ability of *A. thaliana* Hsp81.2 (AtHsp81.2) with SAG1 antigen from *Neospora caninum* in a mouse model of congenital neosporosis. In this case, BALB/c mice i.p. immunized with an equimolar mix of *E. coli*–expressed SAG1 (rSAG1) and rAtHsp81.2 produced a high mixed IgG1:IgG2a response, which could be associated with a balance Th1/Th2- or Th2-type immune response modulation. Besides, after challenge, the immunization with rSAG1 + rAtHsp81.2 mixture increased the protection against vertical transmission of neosporosis in the vaccinated mice and improved the median survival time of offspring (Bengoa Luoni et al., 2019). On the other hand, the authors observed that mice i.p. vaccinated with rNcSAG1 + rAtHsp81.2 or only with rAtHsp81.2 produced high titers of total IgG anti-AtHsp81.2, suggesting that this vaccine formulation would be capable of differentiating vaccinated from infected animals in the field. However, the adjuvant abilities of the parasite Platyhelminthes *Clonorchis sinensis* rCsHsp90 showed that C57BL/6 mice i.p. immunized with a mixture of rCsHsp90 + *E. coli*–expressed proline-rich (ProR) antigen of *C. sinensis* only produced specific IgG1 antibodies (Chung et al., 2017). Although rCsHsp90 assayed as adjuvant elicited a better immune response than Freund's adjuvant, a more detailed analysis showed that there was no specific CTL against ProR in the immunized mice with rCsHsp90 or Freund's adjuvant.

In summary, some authors have demonstrated that Hsp90/gp96 coadministered with the antigen of interest do not generate or generate a very low immune response against it. However, other authors observed an effective immune response against the antigen, highlighting the immunostimulatory capacity of Hsp90/gp96. Considering this, it should be evaluated whether the immunogenic properties of the antigen are playing an important role in the generation of the host's immune response. Furthermore, it would be interesting to evaluate if the peptides + Hsp90 form Hsp90–peptide complexes to conclude if this interaction favors the antigen presentation to pAPCs.

### Heterologous Expression System to Produce Hsp90 as Adjuvant

An important aspect during the evaluation of Hsp90 as adjuvants refers to the source of Hsp90 preparation. Li et al. (2011) expressed full-length Mmgp96 in the yeast *Hansenula polymorpha* (yMmgp96) and compared its adjuvant ability with that observed in *E. coli*–expressed rMmgp96. The biophysical analysis demonstrated that yMmgp96 has similar conformational, peptide-binding, and self-assembly properties compared to mice-derived gp96. Besides, BALB/c mice i.m. immunized with a synthetic HBcAg_87−95_ peptide associated with yMmgp96 (yMmgp96/HBcAg_87−95_ complex) resulted in an increase of CD8^+^ T cells and the activation of specific CTL against HBV infection (Li et al., 2011). Additionally, Ju et al. (2014) observed that the presence of yMmgp96 optimized the formulation of the split H1N1 vaccine as they observed a shift of the immune response toward a Th1 phenotype. This was characterized not only by the presence of neutralizing antibodies and IgG2a antibodies but also by the increase of the number of antigen-specific CD8^+^ T cells and the *in vitro* secretion of IFN-γ and TNF-α (Ju et al., 2014). Interestingly, the challenge with either PFU or PR8, two heterologous H1N1 viruses, revealed that yMmgp96/H1N1 complex–immunized mice had less weight loss, lower mortality rate, less viral titer, specific CTL, and production of IFN-γ. These results confirm the ability of yMmgp96 to confer cross-protection and provide the basis for an Mmgp96-based vaccine for prophylactic and therapeutic applications using the binding complex strategy (Li et al., 2011; Ju et al., 2014). By contrast, rMmgp96 was unable to elicit an effective cellular immune response when it was used as an adjuvant, suggesting that the physicochemical properties of Mmgp96 are not present when used rMmgp96 as an immune stimulator in H1N1 infection model (Li et al., 2011). However, Wang S. et al. (2011) demonstrated the adjuvant capacity of rMmgp96 in the HBV infection model. Also, C57BL/6 mice s.c. immunized with the *E. coli*–expressed HPV16 E7 fused to gp96N (rE7-Xlgp96N_1−355_), elicited a mixed IgG1:IgG2a response, and showed enhanced IFN-γ production (Mohit et al., 2011). Besides, mice immunized with rE7-Xlgp96N_1−355_ and challenged with tumor cells displayed a Th1 immune response strong enough to postpone the tumor growth and to generate potent antitumor effects (Mohit et al., 2011). Moreover, other Hsp90s, such as LiHsp83, AtHsp81.2, or NbHsp90.3, produced in *E. coli*–based expression system, conserved their adjuvant abilities (Rico et al., 1999; Li et al., 2005; Echeverría et al., 2006; Bolhassani et al., 2008; Lee et al., 2011; Mohit et al., 2011; Wang Y. et al., 2011; Chen et al., 2012; Corigliano et al., 2013; Bengoa Luoni et al., 2019; Sánchez López et al., 2019). The adjuvant capacity of Hsp90/gp96 became evident when they are used as purified complexes, fusion protein, or recombinant protein mixture, as well as if they are used as full-length or truncated chaperone versions produced in different expression systems. Therefore, further studies are necessary to elucidate whether an expression system is more advantageous than others and if the high levels of recombinant proteins expression depend on the antigens, the chaperones, or both intrinsic features. In addition to bacterial and yeast expression systems, baculovirus and plant-based expression systems were successful strategies to produce Hsp90 as adjuvants (Albarracín et al., 2015; Zhu et al., 2016). To be noted, the production of recombinant proteins in plants provides many beneficial properties such as scalability, low production costs, and, in the case of vaccine production, absence of pathogenic contaminants (Sander et al., 2019).

## A Proposed Mechanism of Hsp90 Adjuvanticity

pAPCs are the mediators between the innate and the adaptive immune response as immature pAPCs can uptake extracellular antigens and present the chaperoned peptides on MHC II molecules for recognition by CD4^+^ T cells. Additionally, a key feature of pAPCs is the ability to acquire and present exogenous antigens on MHC I in a process also known as “cross-presentation.” Importantly, peptides fused to, bound to, or complexed to Hsp90 or gp96 constitute the source of antigen for pAPC presentation during cross-presentation and cross-priming (Binder and Srivastava, 2005). In addition, extracellular Hsp90s can modulate the host innate immune response regardless of the carried and/or associated peptides, as they can interact and activate antigen-presenting cells (APCs). Because of this feature, these chaperones are very attractive to be studied as immunomodulators, and several authors investigated the immunostimulatory properties of Hsp90 purified from cells or as recombinant proteins, either alone or accompanied with an antigen.

### Hsp90 and Immune Cells Activation

**Figure 1:**
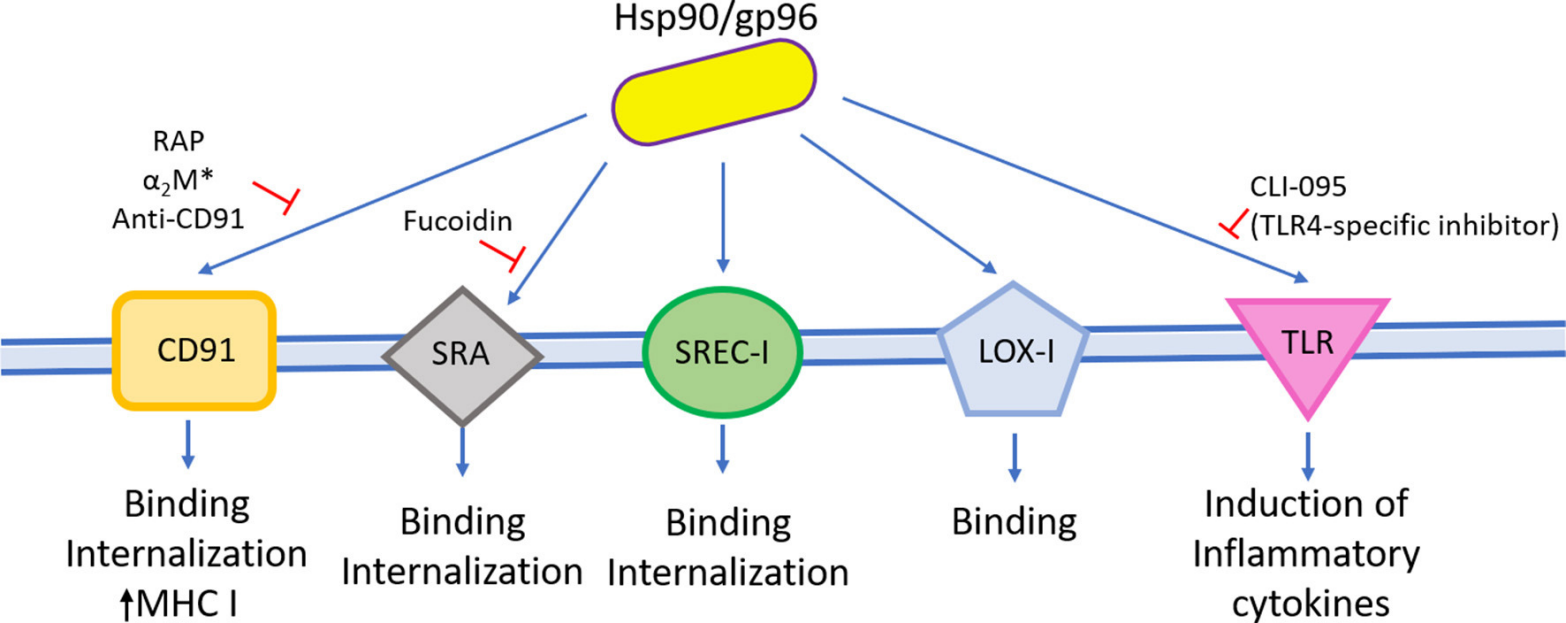
Hsp90 and immune cell activation. Hsp90 and gp96 from several sources can stimulate different cell types involved in the innate immune response and induce the expression of costimulatory molecules and the secretion of cytokines and their proliferation.

Several years ago, Hsp90s were proposed as danger signals because they have two remarkable features: first, they can act as prepackaged danger signal as they constitute 1%−2% of total cellular protein under healthy conditions, and second, they are inducible signals as their synthesis is augmented under several stresses, including heat, cold, irradiation, viruses, bacteria, and even bacterial toxins (Matzinger, 1998). So, when released to the extracellular milieu because of cell damage or necrosis, Hsp90s can bind to the surface receptors present on pAPCs and trigger a proinflammatory immune response (Matzinger, 1998; Basu et al., 2000). In fact, extracellular Hsp90s shape DCs and other cells linked to the immune response by inducing the expression of costimulatory molecules and the secretion of cytokines, as summarized in Table 2 and Figure 1.

**Table 2:** Hsp90 induces cell activation and maturation of different cell types.

**Hsp90 source**	**Hsp90**	**Cell**	**Receptor**	**Maturation**	**Cytokine**	**Nitric oxid**	**Comment**	**References**
*Mus musculus*	Liver-purified Hsp90, gp96	Mouse DC	n.d.	MHC II B7-2	IL-12 IL-1β TNF-α			Basu et al., 2000
*Mus musculus*	gp96	Human DC	n.d.	CD83 CD86	IL-12 TNF-α		gp96 was provided by Immunosome, (Tübingen) but its origin was not indicated	Singh-Jasuja et al., 2000a
		Mouse BMDC	n.d.	CD86 MHC II	IL-12 TNF-α			
*Mus musculus*	Liver-purified gp96	Mouse CD11c^+^ cell	n.d.	CD80, CD86, MHC I, MHC II				Binder et al., 2000a
*Mus musculus*	gp96	BMDC	TLR2 TLR4	CD86	IL-12			Vabulas et al., 2002
*Mus musculus*	Liver-purified gp96	Human myeloid U937 and Raw264.7 cell line at monocyte and macrophage stage	n.d.			Yes		Panjwani et al., 2002
*Leishmania infantum*	*E. coli*-expressed Hsp83	Mouse spleen cells	n.d.				Lymphoproliferation of B cells	Rico et al., 2002
*Mus musculus*	Purified gp96	PMN Monocytes	n.d.		IL-8			Radsak et al., 2003
*Homo sapiens*	Liver-purified gp96	Human immature MDDC	n.d.	CD40, CD83, CD86, HLA-DR				Zhang et al., 2006
	*E. coli*-expressed gp96 N_22−288_, gp96 N_22−336_						Internal regulatory elements are not required for stimulating maturation of MDDC *in vitro*	
	293T cell- expressed gp96 N_22−366_, C_368−799_	Human DC	n.d.	CD86, HLA-DR				
*Mus musculus*	*S. frugiperda-*expressed grp94 N_1−355_	Mouse DC	n.d.	CD40, CD80, CD86, MHC II				Biswas et al., 2006
*Nicotiana benthamiana*	*E. coli*-expressed Hsp90.3	Mouse spleen cells	TLR4				Lymphoproliferation of B cells	Corigliano et al., 2011
*Clonorchis sinensis*	*E. coli*-expressed Hsp90	Mouse MDDC	n.d.	CD40, CD80, CD86, MHC I, MHC II	IL-12 IL-6 IL-1β TNF-α			Chung et al., 2017
*Pseudomonas aeruginosa*	*E-coli-*expressed HtpG	THP-1 at macrophage and monocyte stage	TLR4		IL-8			Lee et al., 2020

*DC, dendritic cells; BMDC, bone marrow-derived dendritic cells; MDDC, monocyte-derived dendritic cells; PMN, polymorphonuclear neutrophils; THP-1, human monocytes cell line; n.d., not determined*.

To study whether this chaperone stimulates the maturation of DCs, Biswas et al. (2006) and Zhang et al. (2006) used gp96 purified from human and mice liver, respectively. Human liver-purified gp96 can partially maturate monocytic-derived DCs (MDDCs) as demonstrated by the increased expression of CD40, CD83, and CD86 molecules (Zhang et al., 2006). Biswas et al. (2006) obtained similar results when using mouse liver-purified gp96. Chung et al. (2017) observed that *E. coli*–expressed *C. sinensis* Hsp90 (rCsHsp90) induce the up-regulation in a dose-dependent manner of costimulatory molecules such as CD40, CD80, CD86, MHC I, and MHC II in mice bone marrow–derived DCs (BMDCs). Besides, they found that BMDCs secreted proinflammatory cytokines (IL-12, IL-6, IL-1β, and TNF-α) in the culture supernatant (Chung et al., 2017). Similarly, murine gp96 induced CD83, CD86, MHC I, MHC II, and B7-2 costimulatory molecule expression, as well as the secretion of IL-1β, IL-12, and TNF-α in immature human DCs and mouse BMDCs *in vitro* (Basu et al., 2000; Binder et al., 2000a; Singh-Jasuja et al., 2000a). Remarkably, Singh-Jasuja et al. (2000a) observed that gp96 did not bind mature DCs, suggesting that mature DCs express reduced levels of the gp96 receptor. Furthermore, Binder et al. (2000a) reported that mice immunized with mouse gp96 elicited an increase of lymph node size. Rico et al. (2002) also showed that *E. coli*–expressed *L. infantum* Hsp83 (rLiHsp83) possess lymphoproliferative abilities. They demonstrated that rLiHsp83 behaves as a B-cell mitogen and that the proliferation of B cells occurs in the absence of adherent and T cells, suggesting a direct binding of rLiHsp83 on B cells (Rico et al., 2002). Interestingly, similar results were obtained by Corigliano et al. (2011) by using nonpathogenic plant Hsp90. They showed that *E. coli*–expressed rAtHsp81.2 and rNbHsp90.3 stimulate the proliferation *in vitro* of naive splenocytes from BALB/c and C3H/HeN mice, and they also determined that plant rAtHsp81.2 and rNbHsp90.3 are mitogens of B cells in an independent T-cell manner (Corigliano et al., 2011).

Besides DCs, macrophages, and B cells, Hsp90s are able to interact with neutrophils and monocytes (Radsak et al., 2003). Fluorescein isothiocyanate (FITC)–labeled mouse-derived gp96 binds to polymorphonuclear neutrophils (PMNs) and monocytes, and the specific binding is interfered with by an excess of unlabeled gp96 and by fucoidan. Panjwani et al. (2002) analyzed whether mouse liver-purified gp96 can activate human myeloid U937 and Raw 264.7 cell line at monocyte and macrophage stages. They observed that nitric oxide is produced by macrophages and DCs in a dose-dependent manner (Panjwani et al., 2002).

Murine gp96 also mediates the release of IL-8 by PMNs and monocytes (Radsak et al., 2003). More recently, Lee et al. (2020) studied the bacterial homolog of Hsp90, also known as HtgG. *E. coli*–expressed HtpG from *Pseudomonas aeruginosa* (rPsHtpG) was able to induce IL-8 secretion in a dose-dependent manner when incubated with THP-1 macrophage and THP-1 monocytes cell lines (Lee et al., 2020). In conclusion, hsp90/gp96 stimulates the host innate immune response.

#### The N-Terminal Region of Hsp90 Has Immunomodulatory Activity

As it was described in previous sections, the N-terminal region of Hsp90/gp96 is strongly associated with the adjuvant capacity of the chaperone. Biswas et al. (2006) studied the mouse N-terminal fragment of glucose-regulated protein (MmGRP94N_1−355_) expressed in *Spodoptera frugiperda*. They showed that MmGRP94N_1−355_ can activate DCs, as demonstrated by up-regulation of CD40, CD80, CD86, and MHC II molecules (Biswas et al., 2006). As well, Zhang et al. (2006) obtained two proteins of the human gp96 N-terminal domain: Hsgp96N_22−288_, which does not contain regulatory elements (that would modulate the association between the N-terminal domain and other domains of gp96), and Hsgp96N_22−336_, which does. Interestingly, *E. coli*–expressed Hsgp96N_22−288_ and Hsgp96N_22−336_ partially activated MDDCs and increased the expression of CD40, CD80, CD86, and HLA-DR molecules, suggesting that the internal regulatory elements are not involved in MDDC maturation *in vitro* (Zhang et al., 2006). On the other hand, they expressed Hsgp96N_22−336_ and the C-terminal fragment of Hsgp96 (Hsgp96C_368−799_) in human embryonic kidney 293T cell line (HEK-293T) derived from HEK-293 cells. Noteworthy, both N- and C-terminal recombinant proteins stimulate DC maturation as indicated by higher expression of CD86 and HLA-DR molecules (Zhang et al., 2006). Considering that the C-terminal domain comprises the last 200 aa (Nemoto et al., 1995), the results obtained by Zhang et al. (2006) also include the Hsgp96 medium domain, and then the immune response observed should not be exclusively assigned to the C-terminal domain.

### Receptors Involved in Exogenous Hsp90 Binding

**Figure 2:**
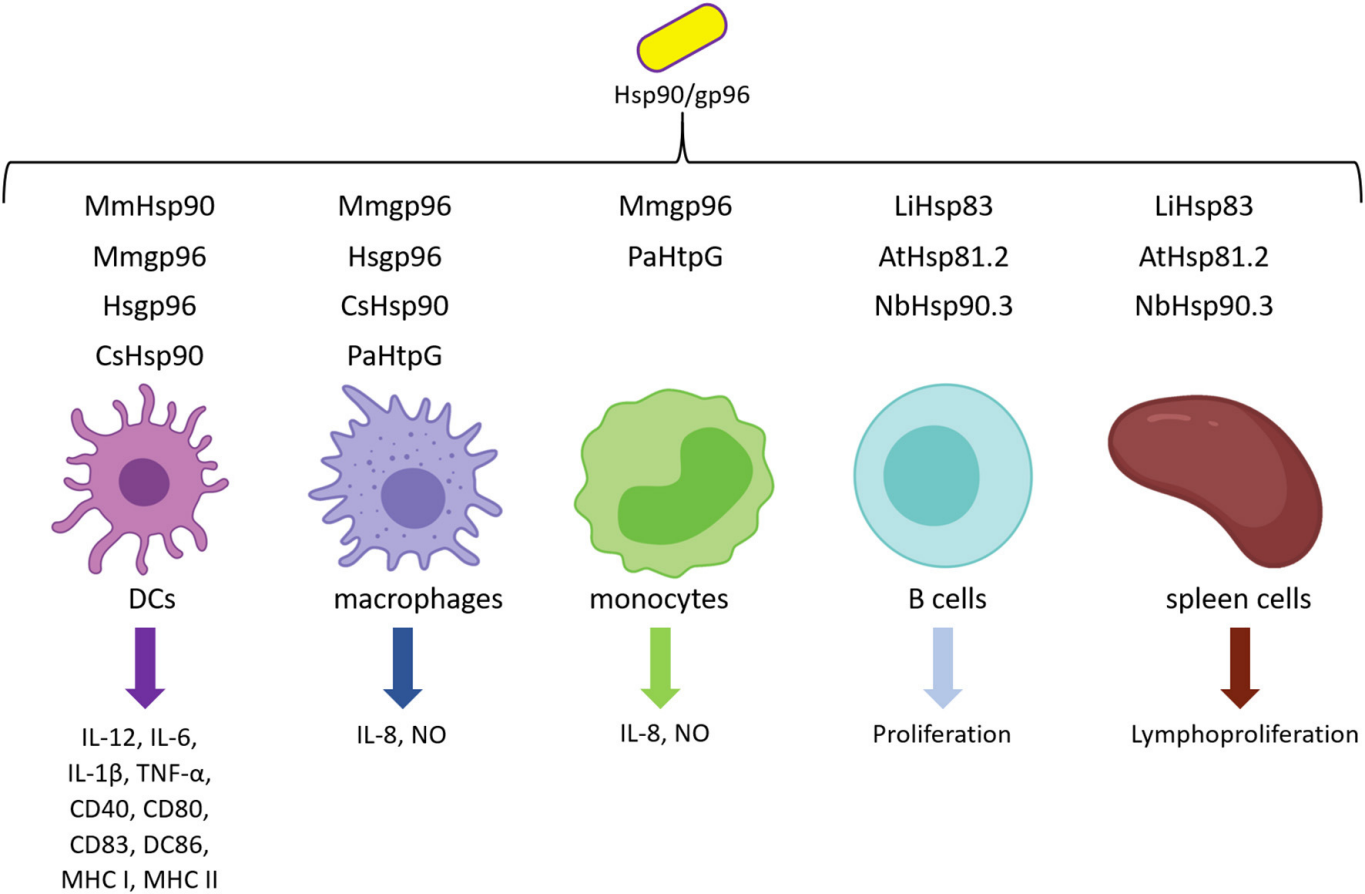
Receptors involved in exogenous Hsp90 binding. CD91, SRA, SREC-I, and LOX-I are receptors reported to bind and internalize exogenous Hsp90. On the other hand, TLR family binds and activates immune cells.

The ability of Hsp90/gp96 to generate robust T-cell response against fused, bound, or mixed peptides result from the specific interaction of these chaperones and professional pAPCs through endocytic receptors. Arnold-Schild et al. (1999) provided the first evidence of receptor-mediated endocytosis of extracellular Hsp90. They observed that murine gp96 labeled with gold particles colocalizes with MHC I molecules in clathrin-coated pits and endosomal structures (Arnold-Schild et al., 1999). Besides, Singh-Jasuja et al. (2000b) showed that FITC-labeled gp96 specifically binds to APC lines, but not to lymphoma cell lines, demonstrating that the binding is mediated by receptors present in pAPCs (Singh-Jasuja et al., 2000b). More importantly, murine gp96 interacts specifically with BMDCs from C57BL/6 and BALB/c mice and human peripheral blood lymphocytes (Singh-Jasuja et al., 2000b). As receptors are internalized very fast after the engagement, Berwin et al. (2002b) performed a binding assay at different temperatures. They observed that red-labeled GRP94 was bound to cell surface receptors at 4°C and internalized via receptor-mediated endocytosis, whereas fluorescein-labeled GRP94 internalized at 37°C via fluid-phase pathway uptake in immature DC was recycled to the extracellular medium within 15 min (Berwin et al., 2002b). After demonstrating specific binding of GRP94 to macrophages surface *in vitro*, Wassenberg et al. (1999) showed that GRP94/peptide can be taken up from the fluid phase, but only receptor-mediated uptake is responsible for directing GRP94 into the pAPCs antigen re-presentation pathway, necessary for CD8^+^ T-cell activation (Wassenberg et al., 1999). Moreover, the internalization efficiency via receptor-mediated endocytosis is higher than a fluid phase process in orders of magnitude (Suto and Srivastava, 1995; Wassenberg et al., 1999; Oura et al., 2011). The evidence presented so far suggests that Hsp90s mediate the cross-presentation process through specific receptors on the surface of pAPCs (Figure 2).

#### Hsp90 and CD91 Receptor

The α_2_-macroglobulin (α_2_M) receptor (CD91) is one of the putative cell surface targets of Hsp90. CD91 is a member of scavenger receptor (SR) family, and it was first proposed as a receptor for gp96 as it was purified as an 80-kDa protein that specifically binds to extracellular murine gp96 (Binder et al., 2000b). Binder et al. (2000b) observed that FITC-coupled gp96 stain RAW 264.7 cells and unlabeled gp96 competed effectively (80% inhibition). Furthermore, they demonstrated that re-presentation of gp96–peptide complexes by MHC I molecules is inhibited by the activated form of the plasma glycoprotein α_2_M (α_2_M^*^) in a concentration-dependent manner. Similar results were obtained by Habich et al. (2002), who performed a competition assay including FITC-labeled human gp96 to J774 A.1 macrophages and observed that α_2_M^*^ and unlabeled gp96 inhibited FITC-gp96 binding in 65 and 56%, respectively (Habich et al., 2002). At the same time, Basu et al. (2001) observed that FITC-α_2_M^*^, murine FITC-gp96, and murine FITC-Hsp90 stained with similar percentages both macrophages from peritoneal cells (90, 97, and 82%, respectively) and RAW 264.7 cell line macrophages (76, 82, and 90%, respectively). As they observed a robust binding and similar staining patterns of gp96, Hsp90, and the CD91 ligand (α_2_M^*^), they concluded that CD91 is a receptor for these chaperones (Basu et al., 2001). However, Berwin et al. (2002a) strongly argued against these results explaining there is no direct evidence of CD91–Hsp90 interaction. By direct analyses of CD91 function, they demonstrated that there is a lack of positive correlation between CD91 and GRP94 at cell surface and during the early trafficking itinerary (Berwin et al., 2002a,b). Besides, these studies showed that either the agonist α_2_M^*^ or the antagonist RAP (receptor-associated protein) of CD91 receptor does not affect GRP94 binding, suggesting that Hsp90/peptide-mediated cross-presentation is independent of CD91 receptor (Berwin et al., 2002a). Furthermore, the SIINFEKL (8-mer) peptide from OVA complexed with GRP94 was re-presented in the presence of α_2_M^*^, suggesting that cross-presentation of GRP94 is CD91-independent (Berwin et al., 2002a). Considering that CD91 internalizes very fast after engaging, Binder and Srivastava (2004) performed the experiments with paraformaldehyde-fixed cells and thus observed that gp96 binds CD91 in a saturable and competitive way, suggesting that it is a receptor for gp96. Indeed, OVA peptides complexed to Hsp90 (gp96/19-mer and gp96/20-mer) are efficiently re-presented *in vitro* and *in vivo*, respectively, and both RAP and α_2_M^*^ inhibit this process (Binder and Srivastava, 2004). The main observation pointed out by Binder and Srivastava (2004) is that they used extended precursors of antigens (OVA 20-mer and OVA 19-mer), which need to be processed before MHC I charging. However, when Binder and Srivastava (2004) repeated the same assay conditions performed by Berwin et al. (2002a), they obtained the same results as Berwin's OVA 8-mer re-presentation assay, suggesting that re-presentation assay directly charges the octapeptide on MHC I molecules, without intracellular processing. Finally, the studies on Hsp90 and CD91 interaction are not restricted to mice model. Robert et al. (2008) observed that *E. coli*–expressed Xlgp96 (rXlgp96) is able to bind to a CD91 homolog at the surface of peritoneal lymphocytes in this amphibian model in a saturable fashion. Also, the addition of non-labeled gp96, α_2_M^*^, RAP, and rabbit anti-CD91 markedly decreases labeled rXlgp96 binding, indicating the interaction of gp96 and CD91 is phylogenetically conserved among amphibians and mammals (Robert et al., 2008). Altogether, these results support to CD91 as a receptor for Hsp90 and gp96.

#### Hsp90 and Scavenger Receptor Family

Besides CD91, other receptors have been reported to bind to exogenous Hsp90, such as SR class-A (SR-A), which is also classified as a member of the C-type lectin family (Berwin et al., 2003). It was demonstrated that the binding surface of mammal gp96 to SR-A present in macrophages and DCs is specific and is partially inhibited by increasing concentrations of unlabeled gp96 and fucoidin, a ligand of SR family (Berwin et al., 2003). These properties were also demonstrated either in HEK-293 cells engineered for expressing SR-A after induction with tetracycline (HEK-SR-A^tet^) or SR-A knockout cells (SR-A^−/−^) (Berwin et al., 2003). Mammal gp96 binding to HEK-SR-A^tet^ was enhanced after induction, but its uptake was abrogated by the addition of an excess of fucoidan. By contrast, SR-A^−/−^ macrophages were impaired to bind and internalize gp96 (Berwin et al., 2003). Also, *in vitro* re-presentation of mammal gp96/8-mer complex on macrophages MHC I demonstrated that SR-A is involved in this process, and the addition of fucoidin inhibited this process in 50% (Berwin et al., 2003). Tewalt et al. (2008) observed that binding and internalization of fluorescent-labeled mammal gp96 to SR-A^−/−^ BMDCs were reduced (68–78%), suggesting the presence of additional receptors in these cells. Also, the addition of fucoidin completely inhibited the binding and internalization of gp96 to background levels, indicating a role for other SR in the uptake of this chaperone (Tewalt et al., 2008). The effector function of CD8^+^ cells assessed by measuring cytolytic activity showed no differences between wild-type and SR-A^−/−^ mice immunized with gp96/OVA 20-mer complex (Tewalt et al., 2008). Finally, Tewalt et al. (2008) proposed that there is a redundancy of Hsp90 receptors as a compensatory mechanism to overcome deficiencies, suggesting that the most likely candidate to compensate the SR-A lack of function is the SR expressed by endothelial cells I (SREC-I) receptor.

SREC-I is an SR class F expressed by immune cells. It was observed that ectopic expression of SREC-I in both RAW 264.7 and CHO cells is sufficient to induce receptor-mediated endocytosis of mammal gp96 (Berwin et al., 2004). Besides, *S. frugiperda*-expressed human Hsp90α complexed to OVA, OVA 13-mer, OVA 8-mer, or OVA 19-mer binds to endogenous SREC-I present on DCs and macrophages or to engineered CHO cells expressing SREC-I (Murshid et al., 2010, 2014). The interaction was confirmed by FACS and fluorescence microscopy in all studied cell types, and it was also demonstrated that this interaction is specific as incubation with an SR competitor diminishes Hsp90α/peptide binding to SREC-I (Murshid et al., 2010). Furthermore, Hsp90α/peptide complex bound to SREC-I cointernalizes in intracellular vesicles in both macrophages and DCs (Murshid et al., 2014), suggesting that it mediates the uptake of Hsp90/peptides complexes into pAPCs.

Finally, the last receptor described to interact with Hsp90 is LOX-I. This is a SR class E that has been also assigned to C-type lectins, which is a large family of receptors characterized by the Ca^2+^-dependent carbohydrate-binding motif. Murshid et al. (2011) observed that *S. frugiperda*–expressed human Hsp90α is able to bind to LOX-I transiently expressed in engineered CHO cells (Murshid et al., 2011).

#### Hsp90 and TLR Receptors

It was demonstrated that maturation and activation of pAPCs are a consequence of Hsp90 binding to Toll-like receptors (TLRs), possibly as a “danger signal” (Basu et al., 2000). Vabulas et al. (2002) observed that BMDCs from mice lacking functional TLR4 and TLR2 receptors, or both do not express CD86 and do not secrete IL-12 in presence of gp96 (Vabulas et al., 2002). Interestingly, similar findings were observed when using plant Hsp90s. rAtHsp81.2 and rNbHsp90.3 stimulated the proliferation of spleen cells from BALB/c and C3H/HeN mice, but they have no effect on C3H/HeJ (TLR4-deficient) spleen cells (Corigliano et al., 2011). Moreover, Lee et al. (2020) demonstrated that TLR4 is involved in the induction of inflammatory cytokines, as they used a TLR4-specific inhibitor (CLI-095) and observed that HtpG-mediated induction of IL-8 decreased in a dose-dependent manner, demonstrating that bacterial Hsp90 is recognized by TLR4. Taken altogether, the immunostimulatory properties of Hsp90s would be, at least in part, mediated by TLR2 and TLR4.

### Hsp90-Mediated Antigen/Peptide Cross-Presentation Mechanism

**Figure 3:**
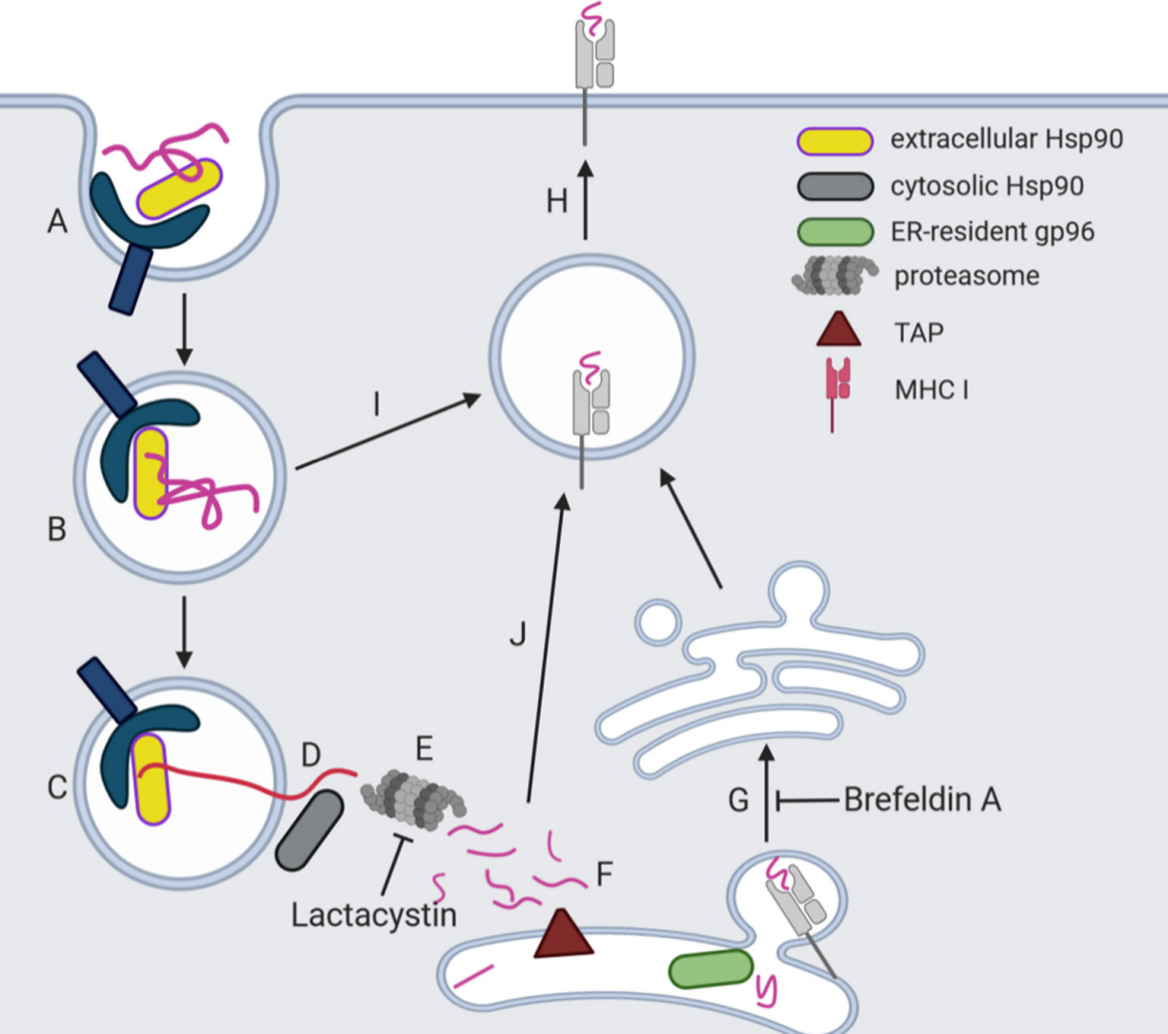
Proposed models for Hsp90-mediated peptide cross-presentation. Cross-presentation process starts after cell surface receptor recognition, binding **(A)** and endocytosis of extracellular/exogenous Hsp90/peptide complexes **(B)**. Three pathways were proposed, and two of them involve peptide translocation (long violet line) from the early endosome to the cytosol for proteasomal degradation **(C)**. There is evidence demonstrating that endogenous Hsp90 (gray) and gp96 are also involved in loading of MHC I with peptides for both endogenous and cross-presentation pathways, although the mechanism is not fully understood **(D)**. To note, extracellular Hsp90 (yellow) also contributes to the translocation of chaperoned peptides from the endosome into cytosol **(D)**. Once in the cytosol, polypeptides are trimmed to small peptides by the proteasome **(E)**. In one model, the digested peptides are imported to the endoplasmic reticulum (ER) by transporter associated with antigen processing (TAP) **(F)**, and peptides are loaded as endogenous antigens **(G)** and then reach cell surface **(H)**. In the other, peptides re-enter into recycling endosomes and load onto MHC I molecules **(J)** and then reach cell surface **(H)**. In the third model, exogenous protein (purple line) is released by Hsp90 (yellow) in early endosomes and charged onto MHC I through a “vacuolar pathway,” probably independent from any cytosolic step **(I)**.

The cross-presentation process conducted by DCs starts after receptor recognition, binding and endocytosis of extracellular/exogenous Hsp90/peptides complexes (Arnold-Schild et al., 1999; Singh-Jasuja et al., 2000b; Kurotaki et al., 2007) (Figure 3A). Several experiments were performed to characterize the mechanism by which Hsp90/peptides complexes can access to MHC I pathway using the OVA and the vesicular stomatitis virus (VSV) antigenic systems. Suto and Srivastava (1995) observed that macrophages pulsed with gp96/VSV20 complexes, but not with gp96 or VSV20 alone, were recognized by VSV-specific CTL. Also, macrophages pulsed with gp96 isolated from EL4 cell transfected with the nucleocapsid protein (N1) of VSV but not those pulsed with gp96 isolated from untransfected EL4 cell released TNF-α and were lysed by VSV-specific CTL in a cytotoxic assay. In addition, Kurotaki et al. (2007) demonstrated that VSV13 or OVA19-mer was taken up by BMDCs and presented in MHC I molecules only when complexed with human Hsp90. Oura et al. (2011) also demonstrated that OVA binding to Hsp90 is essential for peptide cross-presentation. These findings suggest that cross-presentation is mediated by the internalization of exogenous Hsp90s complexed to or fused to a peptide in early endosomes (Figure 3B). Oura et al. (2011), demonstrated that extracellular Hsp90 contributes to the translocation of chaperoned peptides from the endosome into the cytosol by a not well-known mechanism (Figure 3C). However, it was also reported that intracellular Hsp90 is involved in the translocation of exogenous peptides from the endosome to the cytosol (Ichiyanagi et al., 2010; Imai et al., 2011; Kato et al., 2012) (Figure 3D). Once in the cytosol, polypeptides are trimmed to small peptides by the proteasome. Basu et al. (2001) studied the requirement of a functional proteasome complex for the processing and re-presentation of gp96/VSV19 peptide complex *in vitro*. They observed that peritoneal macrophages treated with gp96/VSV19 complex were lysed by VSV8-specific CD8^+^ T cells, and this effect was inhibited by lactacystin, a specific proteasome inhibitor (Basu et al., 2001) (Figure 3E). If the peptides obtained after proteasome cleavage enter to ER, the transporter associated with antigen processing (TAP) pumps peptides into it. Basu et al. (2001) demonstrated that *in vitro* re-presentation of gp96/peptides complex by MHC I requires a functional transporter associated with antigen processing (TAP). They observed that peritoneal macrophages or BMDCs from TAP^+/+^ mice pulsed with gp96/VSV19 complex stimulate specific VSV8 CD8^+^ T cells, whereas APCs from TAP^−/−^ mice were unable to do so (Basu et al., 2001) (Figure 3F). Finally, Suto and Srivastava (1995) used brefeldin A, an inhibitor of protein transport from ER to Golgi. They observed that brefeldin A inhibited the re-presentation of gp96-associated peptides, suggesting that this pathway involves the peptide charge inside the ER (Suto and Srivastava, 1995) (Figure 3G). However, there is evidence showing that exogenous antigens are charged onto MHC I through a “vacuolar pathway,” probably independent from any cytosolic step (Amigorena and Savina, 2010). In this sense, Kurotaki et al. (2007) showed that Hsp90/OVA 13-mer peptide complex is processed, transferred into MHC I within early endosomes, and produced effective cross-presentation. This pathway was studied with BMDCs from TAP^−/−^ mice, and these cells were able to process Hsp90/OVA 13-mer and Hsp90/VSV15 complexes, and present the peptide as efficiently as control BMDCs, suggesting that this process is a TAP-independent process (Kurotaki et al., 2007). It remains elusive if the peptide/antigen is processes by proteases the short peptides charged on MHC I within the recycling endosome (Figure 3I) or if the peptides obtained after proteasome treatment re-enter to the recycling endosome (Figure 3J). Finally, MHC I charged with the peptide reaches the cell surface (Figure 3H). On the other hand, exogenous Hsp90/peptide complex also enters the classical antigen presentation pathway and gets presented by the MHC II molecules (Matsutake et al., 2010; Murshid et al., 2014). Although the re-presentation of exogenous antigen in MHC II is not novel by itself, the novelty derives from the demonstration that re-presentation of Hsp90-chaperoned peptides depends on the interaction of Hsp90s with LOX-I, CD91, and SREC-I (Matsutake et al., 2010; Murshid et al., 2014). Noteworthy, Doody et al. (2004) observed that the resulting CD4^+^ T-cell response did not involve the development of effector functions. To conclude, it appears that Hsp90 could trigger multiple pathways of antigen presentation after binding to surface receptors (Doody et al., 2004).

#### Endogenous Hsp90 Also Participates in Cross-Presentation Mechanism

Interestingly, there is evidence that endogenous Hsp90 is also involved in the loading of MHC I with peptides for both endogenous and cross-presentation pathways. By using Hsp90 inhibitors that block its N-terminal ATP/ADP binding domain, Callahan et al. (2008) demonstrated that radicicol, geldanamycin, and the geldanamycin derivative 17-AAG–treated cells significantly diminished (46, 59, and 48%, respectively) the 4T1 cell surface expression of MHC I when compared to dimethyl sulfoxide control. They concluded that inhibition of Hsp90 affects charging on MHC I with peptides and diminishes the antigen presentation process. Similar results were obtained by Ichiyanagi et al. (2010), who observed inhibition of cross-presentation in BMDCs and DC2.4 cell line in a dose-dependent fashion in the presence of radicicol or 17-AAG. Additionally, Imai et al. (2011) developed Hsp90α^−/−^ mice and unequivocally defined the involvement of Hsp90α function in the direct and cross-presentation pathway. While cross-presentation in Hsp90α^−/−^ BMDCs was significantly down-regulated, the direct presentation was less affected (Imai et al., 2011). Furthermore, radicicol completely blocked residual cross-presentation activity in Hsp90α null BMDCs, suggesting that Hsp90β is also involved in the cross-presentation activity (Imai et al., 2011).

## Conclusions, Future Perspectives, and Challenges

The ability of Hsp90 to chaperone peptides, interact with pAPCs through specific receptors, stimulate pAPCs to secrete inflammatory cytokines, and mediate maturation of DCs, makes them a unique starting point for the generation of the immune response (Basu et al., 2000; Singh-Jasuja et al., 2000a; Vabulas et al., 2002; Chung et al., 2017). For this reason, several studies have been carried out to understand the mechanisms that modulate the immune response mediated by Hsp90. The final goal of this knowledge is to develop different strategies that allow us the use of Hsp90 as adjuvants in the design of vaccines for the prevention of infectious diseases. The ultimate objective of employing Hsp90s as adjuvants and the success of a vaccine formulation will be reached when the selected peptide would be presented on MHC I on pAPCs for CD8^+^ T cell activation.

Cross-presentation triggered by Hsp90 and gp96 complexed or fused to a peptide derived from the ability of these chaperones to bind to endocytic receptors present on surface pAPCs (Berwin et al., 2003; Murshid et al., 2010; Oura et al., 2011). Thus, surface receptor transiently expressed in cells that normally do not express them is a very useful strategy to study Hsp90/gp96 receptor binding. However, the study of the appropriate cell type is not as simple as it seems. Likewise, several receptors have been described to interact with Hsp90 and gp96 *in vitro*, although controversial results and antagonistic conclusions persist because several authors used different strategies in their studies (Suto and Srivastava, 1995; Basu et al., 2001; Berwin et al., 2002a,b; Berwin et al., 2003; Binder and Srivastava, 2004; Kurotaki et al., 2007; Murshid et al., 2010, 2011). The diversity of conditions (cell types, inhibitor concentration, temperature, etc.) in the assays did not allow us to reach definitive conclusions. The contradictory results regarding the role of some receptors (like CD91) in antigen presentation mediated by Hsp90/gp96 demonstrate the complexity of the signaling pathways involved in this mechanism. In this sense, a feature to be considered when studying cross-presentation mechanism is the length of the antigenic peptides, As mentioned above, longer peptides (longer than 13 aa) are recommended because they do not directly charge on MHC molecules on the pAPCs, evidencing the intracellular processing of the peptide (Basu and Matsutake, 2004). This is an important aspect when designing a vaccine using Hsp90/gp96 as a delivery system because a peptide longer than 13 amino acids would be internally processed and appropriately presented on the MHC molecules. However, the pathways through Hsp90/antigen complexes delivered and processed inside the cell were studied, but they still are not well-understood (Arnold-Schild et al., 1999; Singh-Jasuja et al., 2000b; Berwin et al., 2002a; Kurotaki et al., 2007). Contradictory results were obtained related to the dependence of proteasome, TAP, and whether the peptide charge in MHC occurs inside the ER or not (Suto and Srivastava, 1995; Basu et al., 2001; Kurotaki et al., 2007). It is important to mention that endogenous cytosolic Hsp90 and ER-resident gp96 are necessary to accomplish this process (Callahan et al., 2008; Ichiyanagi et al., 2010; Imai et al., 2011). Moreover, the requirement of proteasome to MHC I presentation from Hsp90/peptide has gained support (Berwin et al., 2002b; Callahan et al., 2008). However, when analyzing these evidences altogether makes it evident that the different results observed in pAPC maturation *in vitro* and CTL expansion *in vivo* mediated by Hsp90 and gp96 imply that more complicated mechanisms exist for regulating the immunological activity of these chaperones *in vivo* (Zhang et al., 2006).

Another interesting approach used to study the adjuvanticity of a chaperone is to fuse a peptide in its N- or C-domain. While there is no doubt that the N-terminal domain or N-terminal fragment of Hsp90 and gp96 are potent adjuvants, C-terminal domain or C-terminal fragments remain questionable (Li et al., 2005; Yan et al., 2007; Bolhassani et al., 2008; Mohit et al., 2011; Chen et al., 2012, 2013; Pishraft-Sabet et al., 2015; Zhu et al., 2016, Niu et al., 2016). This is interesting because the region of Hsp90 to be used in the immunization strategies could be limited. Even more, Hsp90 is a large protein, and its heterologous expression can often be hindered. In all the cases reported, the Hsp90–peptide fusion or Hsp90/peptide complex strategy triggered a Th1 immune response accompanied with the generation of IgG2a antibodies, specific CTL, IFN-γ, TNF-α, and protection after pathogen challenge. Interestingly, these results suggest that the Hsp90 dimerization is not involved in the cross-presentation process, and the antigenic peptides can be linked directly to the full-length monomer, the N- or C-terminal of Hsp90 without altering its adjuvant properties. Furthermore, these results show the versatility of Hsp90/gp96 in their use as adjuvants as they are functional irrespective of where the antigenic peptide is inserted.

The Hsp90 fusion strategy to the peptide would guarantee a Th1 immune response, necessary to generate effective protection against infectious diseases, suggesting that this strategy would be the most advantageous in vaccine design against intracellular pathogens. In contrast, the mixture of Hsp90 + peptide not always generates a Th1 immune response. In fact, some studies showed that mice immunized with a mixture Hsp90 + antigen did not provide a specific CTL response, whereas others observed that such mixtures can modulate the humoral and cellular immune response elicited against the antigen (Wang S. et al., 2011; Chen et al., 2013; Niu et al., 2016; Zhu et al., 2016; Chung et al., 2017; Bengoa Luoni et al., 2019). The differences observed in the immune response generated by different antigen formulations could be associated with different intrinsic properties related to the structure and composition of each Hsp90, and this could affect its adjuvant properties. However, further studies will be necessary to elucidate the best way of designing and producing Hsp90 to generate the desired immune response against intracellular pathogens.

As we have discussed throughout this review, numerous studies have shown the potential of using Hsp90 fusion, complex, or mixture vaccine approaches against different intracellular pathogens to enhance both the humoral and cellular immune response. The adjuvant properties of Hsp90 and gp96 were demonstrated in different pathogen models, either in virus, parasite, or bacteria pathogens; the route of administration; the type of vaccine; the dose; or the immunization schedule. Importantly, chaperone adjuvanticity was observed not only in the mice model, but also in pigs and dogs (Chen et al., 2013, Zhu et al., 2016, Niu et al., 2016), and this is encouraging as the development of vaccines for veterinary use is challenging. In addition, different domains or domain fragments were studied to clarify which portion of Hsp90 has adjuvant properties. The challenge in vaccine design is to achieve rational design strategies, which not only involve the selection of the antigens or antigenic peptides but also the most suitable adjuvant to obtain an appropriate immune response and protection. In this sense, the knowledge of the mechanisms involved in the triggering of the immune response mediated by Hsp90 is essential to advance in the design of vaccines using these chaperones as adjuvants. Remarkably, it is conclusive that Hsp90s have unique features that make them worthy adjuvants to be incorporated in vaccine formulations that require the induction of a cell-mediated immune response to prevent infectious diseases.

## Author Contributions

MGC and MC provided the ideas and wrote the draft manuscript. VS and SOA revised the manuscript critically. ES, LM, and VR contributed to the editing and revision of the manuscript. All the authors read the final manuscript.

## Conflict of Interest

The authors declare that the research was conducted in the absence of any commercial or financial relationships that could be construed as a potential conflict of interest.
